# Rice spikelet flowering-state detection based on an improved YOLOv11n model

**DOI:** 10.3389/fpls.2026.1914577

**Published:** 2026-07-27

**Authors:** Hanrui Guo, Hao Wen, Yian Hou, Nan Li, Yingli Cao

**Affiliations:** 1 School of Information and Electrical Engineering, Shenyang Agricultural University, Shenyang, China; 2 National Digital Agriculture Regional Innovation Center (Northeast), Shenyang, China

**Keywords:** ADown module, Convolution–Attention Fusion Module, Detect_Efficient head, hybrid rice breeding, rice spikelet flowering-status detection

## Abstract

Accurate identification of rice spikelet flowering status is critical for germplasm screening in hybrid rice programmes. However, automated detection remains challenging because rice spikelet targets are small, densely distributed, and subject to significant background interference. To address these challenges, this study proposes YOLO11n-ACDW, an improved YOLO11n-based model for rice spikelet flowering-status detection. Specifically, the model incorporates the ADown downsampling module to preserve high-frequency features of small targets, introduces a Convolution–Attention Fusion Module (CAFM) to enhance discrimination between targets and background, adopts the Detect_Efficient detection head to adapt to the scale characteristics of rice spikelet, and integrates the Wise-IoU v3 loss function to improve bounding-box regression accuracy. Experimental results showed that the improved model achieved a precision of 88.8%, a recall of 84.7%, and an mAP@0.5 of 84.0% on the test set, representing improvements of 8.0%, 3.4%, and 1.3% over the baseline YOLO11n, respectively. Compared with the mainstream detector RT-DETR, the proposed model improved these metrics by 9.2%, 6.1%, and 2.5%. In terms of model complexity, it also outperformed the other algorithms, with only 2.14 M parameters and a computational cost of 4.4 GFLOPs, while maintaining lightweight characteristics. These results provide technical support for flowering-window monitoring in hybrid rice breeding.

## 1 Introduction

Rice is fundamental to global food security, and sustained increases in rice yield are essential to meet the challenges posed by population growth and limited resources (Katiyar et al., 2025). As an important approach for improving grain production, hybrid rice exploits heterosis and can achieve yields more than 20% higher than those of conventional rice (Cheng et al., 2007; Cao and Zhan, 2014). Therefore, hybrid rice breeding has become a key strategy for ensuring food security and alleviating the pressures associated with population growth and declining arable land resources. In this context, improving breeding efficiency is critical for the stable enhancement of food supply capacity (Zhang et al., 2026). Accurate identification of rice spikelet flowering status is an essential step in hybrid rice breeding. Although the rice flowering period typically lasts approximately 10 days, each rice spikelet remains in the flowering state for only 1.5–2 hours per day, and pollen remains viable for just 4–5 minutes (Deb et al., 2023; Wang et al., 2023; Ashraf et al., 2024). The brief and highly time-sensitive nature of this process places considerable demands on the accurate detection of spikelet flowering status (Huang et al., 2018; Huang et al., 2026; You et al., 2026).

Existing image-based detection studies in rice and crop phenotyping have mainly focused on medium- to large-scale targets, such as rice panicles, fruits, leaves, weeds, or whole plants (Rai et al., 2023; Tan et al., 2023; Murphy et al., 2024). For example, Xiong et al. combined deep learning with superpixel segmentation to identify rice panicle regions, achieving an accuracy of 76.7% (Xiong et al., 2017). Sun et al. proposed YOLOv8n-SSDW for barnyard grass detection in paddy fields, achieving an mAP@50 of 85.1% (Sun et al., 2025). Object detection models such as Faster R-CNN and RT-DETR have also been applied to fruit counting, panicle identification, and disease detection under complex field backgrounds (Wang et al., 2022; Xiao et al., 2023; Liang et al., 2025; Yan et al., 2025; Wang et al., 2026). Although these methods have demonstrated the potential of deep learning for agricultural phenotyping, their direct transfer to rice spikelet flowering-status detection remains limited. First, when the detection scale is refined from panicles or crop organs to individual spikelet, the extremely small target size and dense distribution substantially weaken feature representation, making it difficult to maintain detection accuracy and stability (Nikouei et al., 2025). Second, rice spikelet targets are easily affected by complex field backgrounds, including leaves, overlapping panicles, soil, and illumination variation, which further increases the difficulty of accurate localization (Khan et al., 2025). Third, most existing models are optimized for general crop organ detection rather than fine-grained flowering-state recognition at the spikelet level.

Studies specifically related to rice spikelet flowering-status detection remain relatively limited. Existing approaches mainly include dynamic flowering monitoring based on traditional visual features, flowering/non-flowering discrimination based on hyperspectral data, and flowering-region detection based on deep learning (Guo et al., 2015; Zhang et al., 2022; Muranaka et al., 2025). Guo et al. used field time-series RGB images combined with SIFT and SVM to achieve flowering-panicle detection and dynamic flowering characterization; however, this method relies on manually designed features and has limited robustness under strong illumination and across different varieties (Guo et al., 2015). Zhang et al. used hyperspectral data combined with RF, SVM, BP, and CNN methods to identify spikelet flowering status; however, this approach depends on specialized spectral equipment and does not directly provide spatial localization in natural field RGB images (Zhang et al., 2022). Therefore, an accurate and lightweight detection method specifically designed for rice spikelet flowering-status monitoring is still needed for practical hybrid rice breeding applications.

Taken together, existing methods for detecting rice spikelet flowering status still require improvement in terms of detection accuracy, small-target representation, and practical deployment efficiency. Therefore, this study aims to develop a lightweight and accurate detection method for rice spikelet flowering-state monitoring under complex field conditions. The main objectives of this study are as follows: (1) to construct a rice spikelet flowering-state detection dataset by combining field image acquisition, image cropping, panicle ROI extraction, and manual annotation correction; (2) to propose an improved YOLO11n-based model, YOLO11n-ACDW, by integrating ADown, the Convolution–Attention Fusion Module (CAFM), Detect_Efficient, and Wise-IoU v3 to enhance small-target feature preservation, target-background discrimination, multi-scale detection, and bounding-box localization; and (3) to systematically evaluate the proposed model through comparative experiments, ablation experiments, visualization analysis, and failure-case analysis, thereby providing technical support for automated flowering-window monitoring in hybrid rice breeding programmes.

## 2 Materials and methods

### 2.1 Data collection

**Figure 1:**
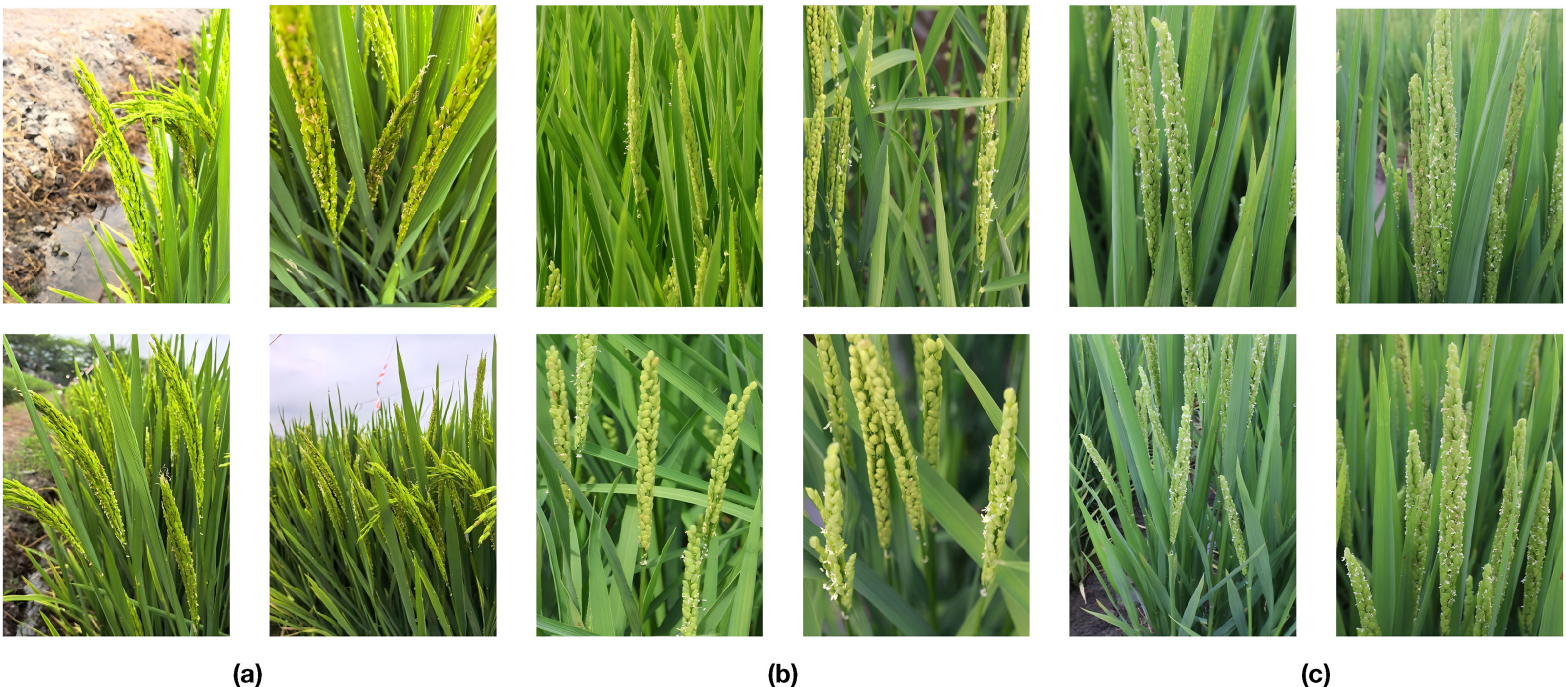
Rice spikelet images from different regions. **(a)** National Seed Breeding Base, Hainan (Apr–May 2025); **(b)** Kalima Rice Experimental Station, Liaozhong (Jul–Aug 2024 & 2025); **(c)** the Rice Research Institute of the Liaoning Academy of Agricultural Sciences, Sujiatun (Jul–Aug 2024 & 2025).

The rice spikelet image data used in this study were collected from experimental fields located in three different regions, as shown in **Figure 1**. Specifically, data were collected from April to May 2025 at the National Seed Breeding Base in Hainan, China (18.33°N, 109.16°E). Additional data were collected from July to August in both 2024 and 2025 at the Kalima Rice Experimental Station in Liaozhong, Liaoning Province, China (41.20°N, 122.38°E), and at the Rice Research Institute of the Liaoning Academy of Agricultural Sciences (41.83°N, 123.58°E). Sanya, Hainan Province, has a tropical maritime monsoon climate and is mainly used for the breeding and cultivation of different parental materials and combinations of hybrid rice. The Liaozhong Kalima Rice Experimental Station and the Rice Research Institute of the Liaoning Academy of Agricultural Sciences are located in a temperate semi-humid continental climate zone and include diverse breeding materials, such as northern japonica hybrid rice and indica–japonica hybrids.

Data collection primarily focused on rice spikelet targets at the flowering stage, covering two typical states: the initial flowering stage, in which the glumes were slightly open and the anthers were not exposed, and the full flowering stage, in which the glumes were fully open and the anthers were exposed and releasing pollen.

**Figure 2:**
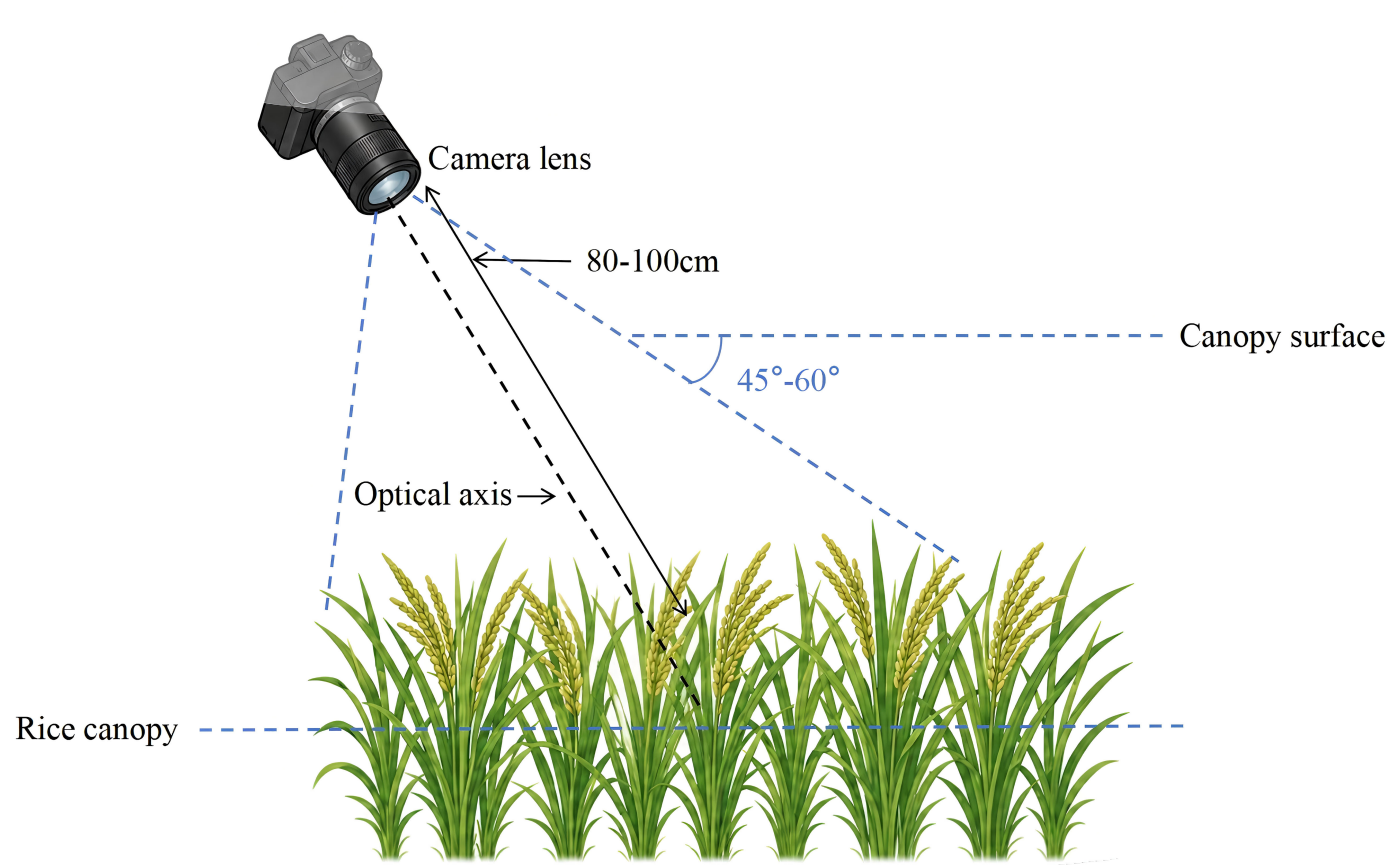
Schematic diagram of field image acquisition.

This study used a Canon EOS-R7 mirrorless camera and an Apple iPhone 16 Pro Max smartphone as the main image acquisition devices. Data collection was conducted daily from 9:30 a.m. to 3:00 p.m. (Beijing time), covering the main flowering period of rice spikelet. All images were acquired under natural light and included two typical illumination conditions, namely sunny and cloudy weather. All images were captured at an approximate distance of 80–100 cm from the rice canopy, with an oblique downward shooting angle of approximately 45°- 60° relative to the canopy surface. No optical zoom was used during image acquisition. A schematic diagram of the field image acquisition procedure is shown in **Figure 2**. To maintain consistency in relative image scale, the shooting distance, viewing angle, and field of view were kept as consistent as possible during field image acquisition. In terms of the sampling strategy, a completely randomized method was used to select healthy rice plants at the flowering stage in each experimental field. For each sampled plant, images of spikelet on both the main panicle and tiller panicle were collected to ensure that the samples adequately reflected the flowering status of individual plants. A total of 3632 raw images were obtained, including 1218 from Sanya, Hainan, 1156 from the Liaozhong Experimental Station, and 1258 from the Liaoning Academy of Agricultural Sciences. After preliminary screening to remove blurred, overexposed, out-of-focus, and duplicate images, 3286 valid images were retained for subsequent dataset construction and model training (**Table 1**).

**Table 1:** Data sources and distribution of valid images and labels.

Address	Number of images	Number of labels
National seed breeding base in Hainan, Sanya	1011	5846
Kalima Rice Experimental Station, Liaozhong	1050	4096
Rice Research Institute of the Liaoning Academy of Agricultural Sciences	1225	2560
Total	3286	12502

### 2.2 Data annotation

Given that each rice spikelet target is only 2–3 mm in diameter, they often account for less than 1% of the pixel area in an image. In addition, a single panicle may contain 50–200 rice spikelet that are densely distributed and easily affected by complex backgrounds such as soil, causing target features to be overwhelmed by background information during feature extraction (Parida et al., 2022; Yan et al., 2022; Deng et al., 2024). Although YOLO-series models rely on multi-scale feature representation and saliency learning of target regions, when the proportion of small targets is extremely low, shallow features are highly susceptible to downsampling and background noise, resulting in reduced representation capability (Han et al., 2025). Therefore, this study adopted a progressive data processing strategy consisting of image segmentation, panicle region-of-interest (ROI) extraction, and spikelet annotation. In this framework, panicle ROI extraction was performed based on the efficient segmentation capability of the SAM model, which substantially reduced the manual workload, while subsequent manual correction further improved the reliability of region extraction and the consistency of data annotation. This strategy enabled the model to simultaneously learn panicle-level spatial structural information and spikelet-level fine-grained features, thereby improving the discriminability of foreground targets during feature learning and providing more discriminative input representations for the subsequent detection network.

The specific processing workflow was as follows. First, the collected raw field images were divided into fixed-size grids and cropped into non-overlapping square sub-images of 1280 × 1280 pixels, while blurred images and images containing invalid content were removed. This grid-based cropping strategy reduced the local distribution density of spikelet and alleviated the problem that small target features could be overwhelmed by complex background information. To reduce potential correlation between data subsets and prevent data leakage, all sub-images derived from the same raw image were assigned to the same subset. The filtered valid sub-images were then divided into mutually exclusive training, validation, and test sets at a ratio of 7:2:1. All subsets were kept fully independent throughout the entire process. In this study, SAM was integrated into the LabelMe platform as an interactive annotation-assistance tool for semi-automatic panicle ROI extraction. For the cropped sub-images in each subset, SAM was used to generate candidate masks of rice panicle regions through point-based prompts. The generated candidate masks were then carefully reviewed and manually corrected in LabelMe to ensure the reliability of the final panicle ROI images. This approach helped highlight the foreground regions containing spikelet targets and reduce background interference during ROI preparation. Subsequently, using LabelMe software, flowering rice panicles were annotated on the basis of the panicle ROI images and their corresponding original sub-images, and the annotations were saved as.txt label files. The rice flowering panicle detection dataset used for model training and evaluation was constructed from panicle ROI images and the corresponding original sub-images at a ratio of 1:1.

## 3 Construction of the YOLO11n-ACDW model

**Figure 3:**
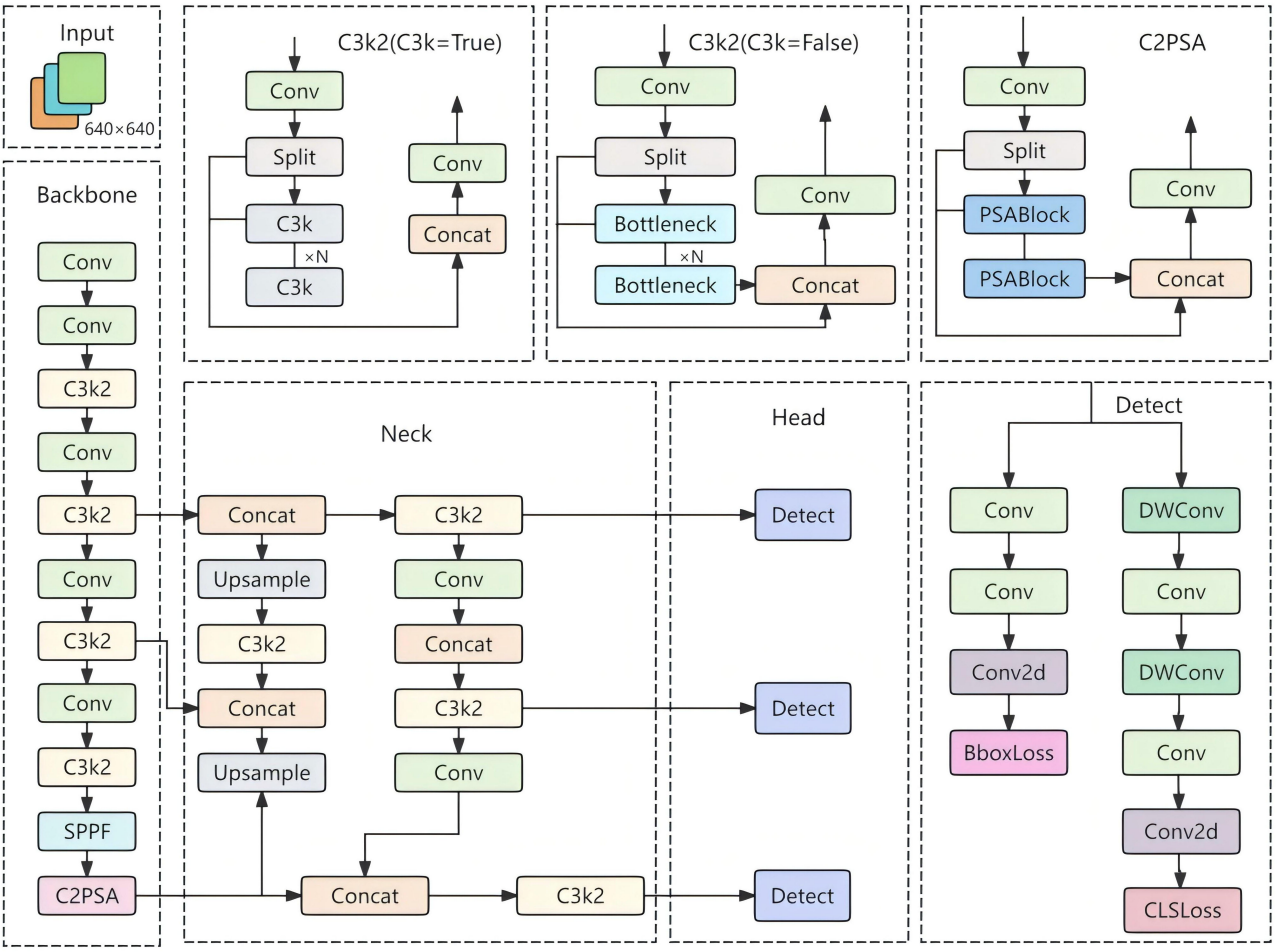
YOLO11n network architecture.

YOLO11, proposed by the Ultralytics team, is one of the representative object detection models developed in recent years that balances detection accuracy and computational efficiency. The overall architecture of YOLO11 is shown in **Figure 3**. This series has been systematically optimized in terms of network architecture design, parameter scale control, and inference efficiency, and provides multiple model-size configurations for different computational conditions. Among them, YOLO11n offers the advantages of a small number of parameters, low computational complexity, and fast inference speed, making it suitable for deployment on field terminals and embedded devices with limited computational resources (Jocher and Qiu).

**Figure 4:**
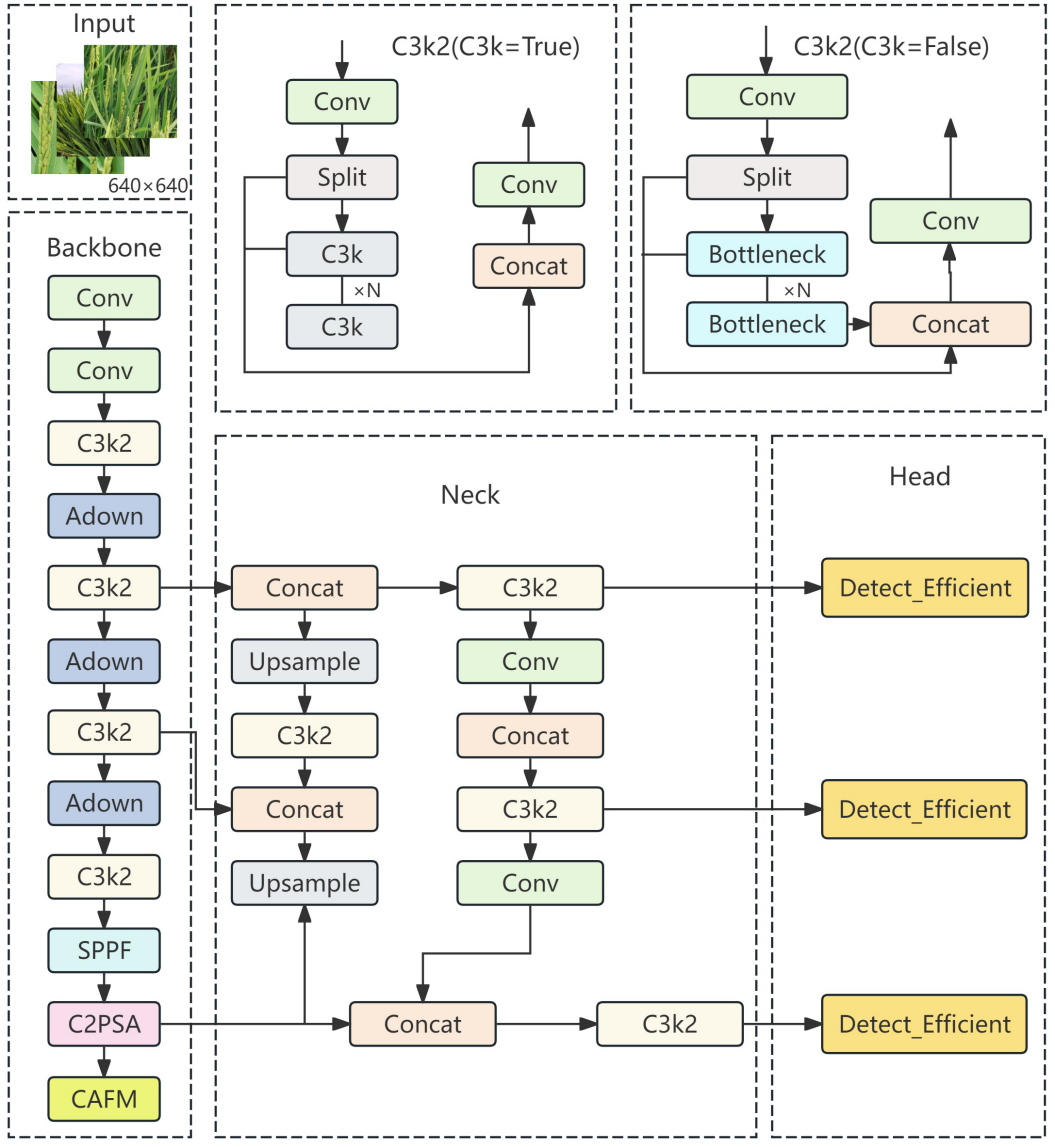
YOLO11n-ACDW network architecture.

However, under complex field conditions, the detection of rice flowering panicles still faces challenges, including weak small-target features, strong background interference, and insufficient localization accuracy. The architecture of the proposed YOLO11n-ACDW model is shown in **Figure 4**. To address these problems, this study developed YOLO11n-ACDW based on YOLO11n as the baseline and introduced improvements in four aspects: downsampling, feature enhancement, detection head design, and loss function optimization. Specifically, the ADown module was introduced to alleviate the loss of fine-grained information during feature compression, the CAFM module was embedded to enhance the response to key panicle regions, the Detect_Efficient detection head was introduced to improve the representation and localization of dense targets, and the Wise-IoU loss function was adopted to optimize bounding-box regression quality.

### 3.1 Design of a detail-preserving downsampling method for tiny spikelet targets

In the task of detecting rice spikelet flowering status, the targets typically exhibit the characteristics of small size, dense distribution, and complex spatial structure. Although traditional downsampling methods based on strided convolution can effectively reduce feature map resolution and computational cost, they often inevitably weaken key detailed features, such as spikelet edges and anther exposure, while compressing spatial dimensions, thereby adversely affecting the accurate localization and discrimination of flowering panicles in subsequent stages.

**Figure 5:**
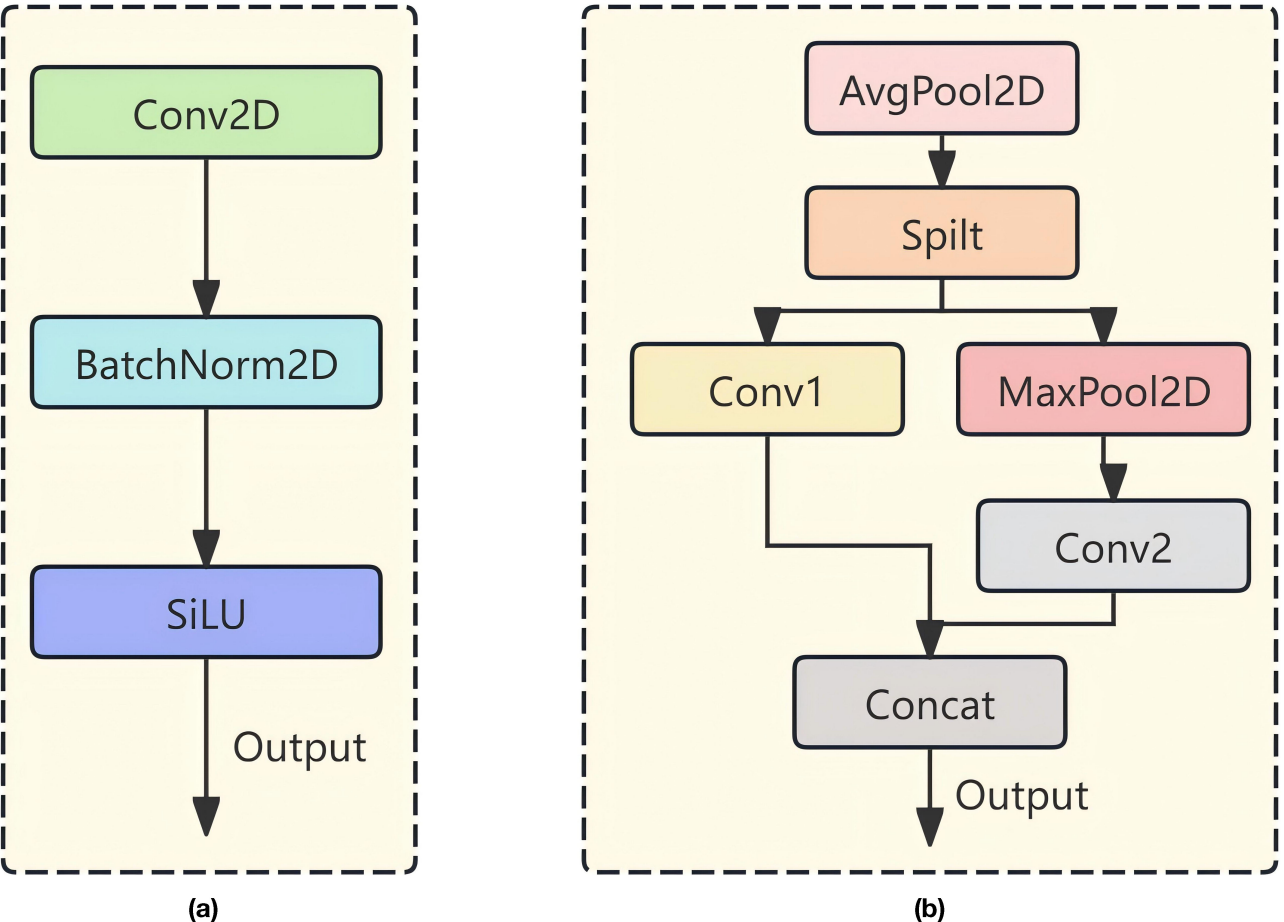
Comparison of Conv downsampling and ADown downsampling structures. **(a)** Conv downsampling; **(b)** ADown downsampling.

To address the above problems, this study introduced the ADown downsampling module into the downsampling stage of the YOLO11n backbone network to replace part of the traditional stride-convolution structure, thereby enabling targeted optimization of the feature downsampling process. Through multi-path feature processing and channel-level reorganization, the ADown module reduces the spatial resolution of feature maps while preserving, to the greatest extent possible, local response information closely related to the discrimination of rice flowering panicles. Its structural diagram is shown in **Figure 5**.

Let the input feature map be defined as X∈RCin×H×W. As shown in Equation 1, the ADown module first applies average pooling with a kernel size of 2×2, stride of 1, and padding of 0 to perform preliminary feature smoothing and spatial alignment:

(1) $$X_{avg}=AvgPool_{2\times 2,s=1,p=0}\left(X\right)$$

According to Equation 2, the pooled feature map is then split into two branches along the channel dimension:

(2) $$X_{1},X_{2}=Split\left(X_{avg}\right)$$

Where X_1_ and X_2_ denote the two channel-wise feature branches. For the first branch, a 3×3 convolution with a stride of 2 and padding of 1 is used to perform convolutional downsampling, as shown in Equation 3:

(3) $$Y_{1}=Conv_{3\times 3,s=2,p=1}\left(X_{1}\right)$$

For the second branch, max pooling with a kernel size of 3×3, stride of 2, and padding of 1 is first applied to enhance local salient responses, as shown in Equation 4:

(4) $$X_{max}=MaxPool_{3\times 3,s=2,p=1}\left(X_{2}\right)$$

The pooled feature is then processed by a 1×1 convolution with a stride of 1 and padding of 0 for channel adjustment, as shown in Equation 5:

(5) $$Y_{2}=Conv_{1\times 1,s=1,p=0}\left(X_{max}\right)$$

Finally, the outputs of the two branches are concatenated along the channel dimension to obtain the downsampled output feature map, as shown in Equation 6:

(6) $$Y=Concat\left(Y_{1},Y_{2}\right),\;Y\in R^{C_{out}\times \frac{H}{2}\times \frac{W}{2}}$$

Where Cout denotes the output channel number after channel adjustment. In this design, the first branch performs convolutional downsampling to extract structural information with a certain spatial receptive field, whereas the second branch uses max pooling to retain salient local responses, followed by 1 × 1 convolution for channel alignment. The final concatenation fuses the two complementary feature representations.

Compared with standard downsampling structures, the ADown module effectively alleviates the problem of excessive smoothing of small spikelet targets in deep features while compressing the feature scale, allowing the model to continue perceiving the distribution characteristics and spatial variation of flowering spikelet within the panicle in subsequent network layers. This characteristic is particularly important for detection tasks in which rice spikelet occupy only a small proportion of the image but play a decisive role in flowering-status discrimination.

By introducing the ADown module during the feature downsampling stage, the model can suppress redundant background features to a certain extent at an early stage and enhance its ability to represent key information of small-scale flowering panicles. This provides a more stable and discriminative feature foundation for subsequent attention guidance and precise regression by the detection head.

### 3.2 A feature enhancement and fusion method for complex field backgrounds

**Figure 6:**
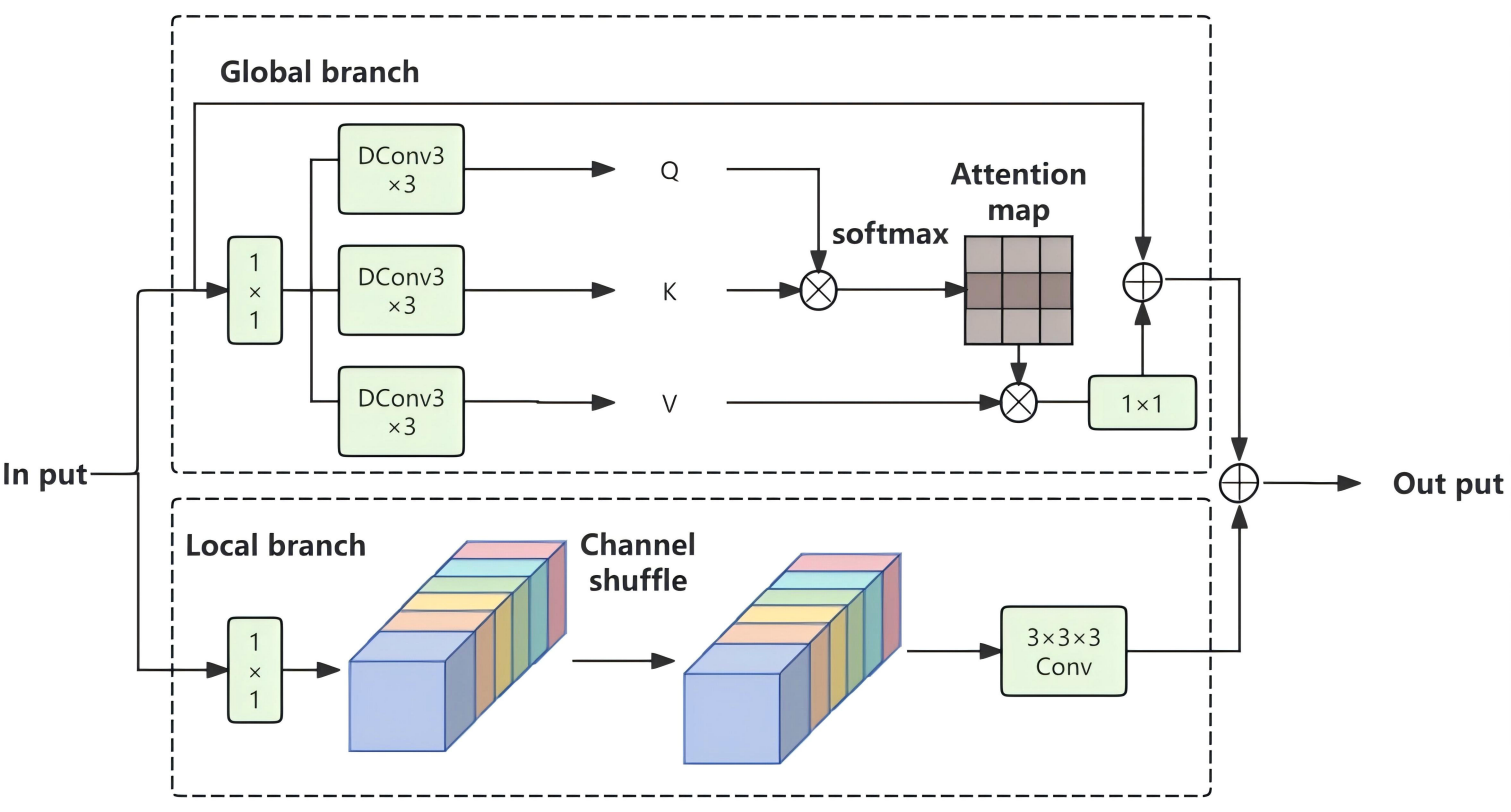
Structure of the CAFM module.

In complex field environments, flowering rice panicles are often intertwined with non-target structures such as leaves and rachises, resulting in substantial background interference and causing the model to produce excessive responses to irrelevant regions. The architecture of the CAFM module is shown in **Figure 6**. To enhance the model’s selective modeling capability for task-relevant features, this study introduces a Convolution–Attention Fusion Module (CAFM) into the YOLO11n model. Through the coupled interaction between the global attention branch and the local convolution branch, this module achieves information alignment and dynamic weighting across feature branches.

Given an input feature tensor $\text{X}\in {\text{R}}^{\text{C}\times \text{H}\times \text{W}}$, CAFM first performs channel projection using 1×1 convolutions to obtain the input features for the global and local branches, as shown in Equation 7:

(7) $${\text{X}}_{\text{g}}={\text{Conv}}_{1\times 1}(\text{X}),\;{\text{X}}_{\text{l}}={\text{Conv}}_{1\times 1}(\text{X})$$

Where Xg and X_l_ denote the projected features of the global branch and local branch, respectively. In the global branch, three independent 3×3 depthwise separable convolutions are used to generate the query tensor Q, key tensor K, and value tensor V, as shown in Equation 8:

(8) $$\text{Q}=\text{D}{\text{Conv}}_{3\times 3}^{\text{Q}}\left({\text{X}}_{\text{g}}\right),\text{ K}=\text{D}{\text{Conv}}_{3\times 3}^{\text{K}}\left({\text{X}}_{\text{g}}\right),\text{V}=\text{D}{\text{Conv}}_{3\times 3}^{\text{V}}\left({\text{X}}_{\text{g}}\right)$$

Where DConv_3×3_ denotes the 3×3 depthwise separable convolution. The attention operation is then formulated as shown in Equation 9:

(9) $${\text{F}}_{\text{att}}=\text{VSoft}\max\left(\frac{\text{KQ}}{\alpha }\right)$$

Where α is a learnable scaling parameter used to control the distribution of attention weights before the Softmax operation. Compared with conventional attention mechanisms using a fixed scaling factor, the learnable parameter enables the attention branch to adaptively adjust the attention distribution according to feature characteristics.

After attention weighting, as shown in Equation 10, the attention-enhanced feature is fused with the projected global feature through a residual connection and then reorganized by a 1×1 convolution:

(10) $${\text{F}}_{\text{g}}={\text{Conv}}_{1\times 1}\left({\text{F}}_{\text{att}}+{\text{X}}_{\text{g}}\right)$$

Where Fg represents the output feature of the global attention branch. This branch enhances the representation of panicle regions associated with flowering status at the global scale while suppressing irrelevant responses from leaf texture, rachis interference, and complex background noise.

In the local branch, the projected feature Xl is processed by channel shuffle to promote cross-channel information interaction. Then, a 3×3×3 convolution is used to further extract fine-grained local structural features, such as spikelet edges, dense spikelet arrangements, and anther-related textures.

(11) $${\text{F}}_{\text{l}}={\text{Conv}}_{3\times 3\times 3}\left(\text{CS}\left({\text{X}}_{\text{l}}\right)\right)$$

As defined in Equation 11, Where F_l_ represents the output feature of the local branch, and CS denotes the channel shuffle operation. This branch strengthens the representation of tiny and densely distributed spikelet, which is critical for discriminating flowering status under occlusion and overlap conditions.

Finally, as shown in Equation 12, the global attention feature Fg and the local convolutional feature Fl are fused by element-wise addition to obtain the final output of the CAFM module:

(12) $${\text{F}}_{\text{out}}={\text{F}}_{\text{g}}+{\text{F}}_{\text{l}}$$

Where F_out_ denotes the fused output feature of CAFM. Through this convolution–attention fusion strategy, the module coordinates local details and global contextual information. The global branch captures long-range dependencies and suppresses background interference, while the local branch preserves fine-grained structural details of rice panicle and spikelet. Therefore, the fused features can simultaneously maintain global semantic consistency and local structural discrimination, which is beneficial for detecting tiny and densely distributed spikelet in complex field environments.

In conjunction with the preceding data processing strategy, this module extends the design concept of emphasizing panicle features while suppressing background interference at the feature level. This facilitates the stable preservation of information highly correlated with flowering status during multi-scale fusion, thereby improving the model’s detection robustness and generalization ability in complex field environments.

### 3.3 An optimized multiscale detection method for densely distributed spikelet

During the target prediction stage, the original YOLO11n detection head still shows limited capability in multi-scale response and feature allocation when handling flowering panicles with slender structures, dense distribution, and dispersed scales, which has become a factor limiting further improvement in overall detection performance.

In the task of detecting rice spikelet flowering status, the main detection target is the flowering panicle, whose scale in images typically lies between that of conventional small and medium-sized targets. In addition, differences in growth stage, shooting distance, and viewing angle lead to a certain degree of dispersion in panicle scale. Although the original YOLO11n detection head has multi-scale feature fusion capability, when dealing with targets characterized by slender structures and dense distribution, it tends to show insufficient multi-scale response efficiency, thereby leading to missed detections.

**Figure 7:**
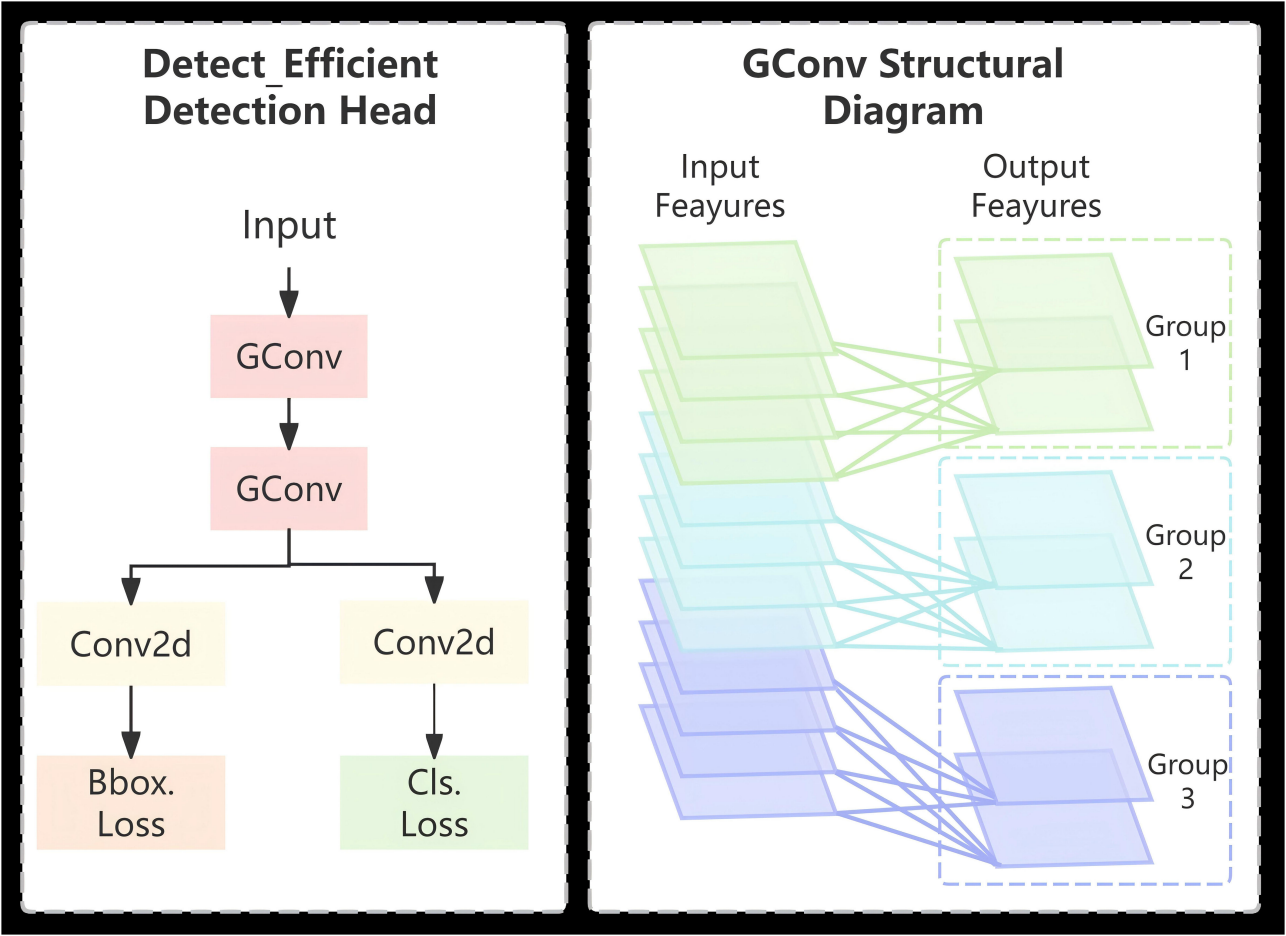
Detection head structure.

This study introduces the Detect_Efficient detection head into the YOLO11n model to achieve targeted optimization at the object prediction stage. The architecture of the Detect_Efficient detection head is shown in **Figure 7**. By improving the prediction-branch structure and scale-response mechanism, this detection head enables the model to adapt more flexibly to the scale variations of flowering panicles across different scenarios, thereby enhancing localization stability and discrimination capability for panicle targets. Compared with the original detection head, Detect_Efficient places greater emphasis on response efficiency at task-relevant scales, effectively alleviating the problem of information dilution caused by uneven feature allocation.

Let ${\text{F}}_{\text{i}}\in {\text{R}}^{{\text{C}}_{\text{i}}\times {\text{H}}_{\text{i}}\times {\text{W}}_{\text{i}}}$ denote the input feature map at detection scale i, where i∈{1,2,3}. As shown in Equation 13, In Detect_Efficient,lightweight feature transformation is performed using stacked group convolutions:

(13) $${\hat{\text{F}}}_{\text{i}}=\text{GCon}{\text{v}}_{3\times 3}\left(\text{GCon}{\text{v}}_{3\times 3}\left({\text{F}}_{\text{i}}\right)\right)$$

Where ${\hat{F}}_{i}$ denotes the transformed feature map at detection scale i, and GConv_3×3_ denotes the 3×3 group convolution. As defined in Equation 14, the classification and bounding-box regression outputs are generated and supervised by the corresponding loss branches during training:

(14) $${\text{O}}_{\text{i}}^{\text{cls}}=\text{Con}{\text{v}}^{\text{cls}}\left({\hat{\text{F}}}_{\text{i}}\right),\;{\text{O}}_{\text{i}}^{\text{box}}=\text{Con}{\text{v}}^{\text{boc}}\left({\hat{\text{F}}}_{\text{i}}\right)$$

(15) $${\text{O}}_{\text{i}}=\text{Concat}\left({\text{O}}_{\text{i}}^{\text{cls}},{\text{O}}_{\text{i}}^{box}\right)$$

According to Equation 15, ${\text{O}}_{\text{i}}^{\text{cls}}$ and ${\text{O}}_{\text{i}}^{\text{box}}$ represent the classification output and bounding-box regression output, respectively, and O_i_ denotes the final prediction output at detection scale i.

This detection head replaces standard convolutions with group convolutions and simplifies the feature extraction process before the classification and regression branches, thereby improving parameter sharing and reducing computational cost. GConv limits dense inter-channel connections through group constraints. This structure not only markedly decreases the number of module parameters but also lowers computational complexity, making it advantageous for deployment on edge devices. The parameter relationship between standard convolution and group convolution is given in Equation 16:

(16) $${\text{N}}_{\text{GConv}}=\frac{1}{\text{G}}{\text{N}}_{\text{Conv}},\;{\text{N}}_{\text{Conv}}={\text{C}}_{\text{in}}{\text{C}}_{\text{out}}{\text{K}}^{2}$$

Where G is the number of groups, K is the convolution kernel size, and C_in_ and C_out_ denote the numbers of input and output channels, respectively. This indicates that group convolution reduces the parameter number to approximately 1/G of that of standard convolution under the same channel settings.

### 3.4 An optimized bounding-box regression method for fuzzy spikelet boundaries

The YOLO11n model uses CIoU (Complete Intersection over Union) as the bounding-box loss function. Compared with the traditional IoU loss, CIoU incorporates geometric factors such as the position, distance, and shape of bounding boxes, thereby further improving the accuracy and convergence speed of bounding-box regression. However, because it requires additional calculations of complex parameters, such as the distance between bounding boxes and aspect ratios, its computational cost is relatively high. In addition, in the detection task of this study, targets are easily affected by leaf occlusion and panicle overlap, resulting in a large number of low-quality samples in the dataset that are difficult to annotate accurately. Under these conditions, the strong penalty introduced by geometric factors may cause the model to focus excessively on difficult samples while neglecting effective learning from ordinary samples.

To this end, this study introduces the Wise-IoU v3 loss function into the model to replace the traditional CIoU loss for bounding-box regression optimization. Wise-IoU v3 is extended from Wise-IoU v1 by introducing a non-monotonic focusing coefficient, and its loss is defined in Equation 17:

(17) $$L_{WIoUv3}=\frac{\beta }{\delta \alpha^{\beta -\delta }}L_{WIoUv1}$$

(18) $$\beta =\frac{{\text{L}}_{\text{IoU}}}{\bar{{\text{L}}_{\text{IoU}}}}$$

As defined in Equation 18, β denotes the outlierness of a prediction box, with a smaller β value indicating higher prediction-box quality. α and δ are two adjustable parameters used to control the mapping relationship between the non-monotonic focusing coefficient and β. This outlierness metric directly links prediction-box quality to the IoU loss: prediction boxes with larger localization errors correspond to higher β values, whereas high-quality prediction boxes have β values approaching 0.

On this basis, Wise-IoU v3 achieves sample-adaptive regression through a dynamic gradient-gain mechanism. The loss of the Wise-IoU v1 base module is computed according to Equation 19, where the IoU term is coupled with a dynamic weight term.

(19) $$L_{WIoUv1}=R_{WIoU}L_{IoU}$$

(20) $$R_{WIoU}=\exp\left(\frac{{\left(x_{a}-x_{b}\right)}^{2}+{\left(y_{a}-y_{b}\right)}^{2}}{W^{2}+H^{2}}\right)$$

As defined in Equation 20, R_WIoU_ is a dynamic weighting term based on the center offset between bounding boxes, which modulates the gradient gain through an exponential function. When the center offset between the predicted box and the ground-truth box is large, R_WIoU_ approaches 1, thereby suppressing the gradient contribution. When the center offset is small, R_WIoU_ approaches e^−1^ thereby enhancing the gradient contribution. This dynamic modulation enables the model to focus more on difficult samples with large localization errors during training, while preventing high-quality samples from overly dominating gradient updates, thereby maintaining convergence stability even under conditions of uneven annotation quality.

Furthermore, while retaining the IoU constraint form, Wise-IoU v3 introduces a center-offset-related weighting term, R_WIoU_ to strengthen the penalty on bounding-box center-position errors without increasing computational complexity, thereby compensating for the limited ability of traditional IoU-based losses to model spatial relationships.

### 3.5 Experimental environment and evaluation metrics

To ensure the comparability of experimental results across different models, all comparative experiments were conducted under identical software and hardware conditions. The software environment comprised Python 3.8.18, PyTorch 2.2.1 as the deep learning framework, and CUDA 11.8 for acceleration. All experiments were performed on an NVIDIA GeForce RTX 4060 Ti GPU with 16 GB of memory. During training, the YOLO-series models were configured with a unified input image size of 640 × 640, a batch size of 32, and 300 epochs. The SGD optimizer was used, with an initial learning rate of 0.01 and a weight decay of 0.0005. These standardized settings were adopted to ensure the fairness of the comparative experiments and to minimize performance variations caused by differences in training conditions. To ensure proper convergence and optimal performance, Faster R-CNN and RT-DETR were trained according to the hyperparameter settings recommended in their official implementations. These settings were adopted to guarantee a fair comparison while minimizing the influence of differences in training conditions on the experimental results.

To comprehensively evaluate the detection performance and engineering applicability of the model for rice flowering panicle detection, this study established an evaluation framework from two aspects: detection accuracy and model complexity. Considering the characteristics of the field environment, including small target size, complex backgrounds, and the coexistence of missed detections and false detections, precision (P), recall (R), and mean average precision at an IoU threshold of 0.5 (mAP_0.5_) were selected as the main detection performance metrics. In addition, the number of parameters, computational cost (GFLOPs), and model weight were introduced to evaluate the lightweight characteristics of the model.

#### 3.5.1 Precision and recall

Precision is used to measure the proportion of samples predicted as flowering panicles that are truly positive, reflecting the model’s ability to suppress background interference. It is calculated according to Equation 21:

(21) $$\text{P}=\frac{\text{TP}}{\text{TP}+\text{FP}}$$

Here, TP denotes the number of flowering panicles correctly detected, and FP denotes the number of non-target samples incorrectly classified as flowering panicles.

Recall is used to measure the proportion of true flowering panicle targets that are successfully detected, reflecting the model’s ability to control missed detections. It is calculated according to Equation 22:

(22) $$\text{R}=\frac{TP}{TP+FN}$$

Here, FN denotes the number of true flowering panicle targets that were not detected.

#### 3.5.2 Mean average precision

Mean average precision (mAP) is a commonly used comprehensive evaluation metric in the field of object detection and can reflect the overall detection performance of a model under different confidence thresholds. In this study, mAP at an IoU threshold of 0.5, denoted as mAP_0.5_, was used as the primary accuracy evaluation metric.

First, the Intersection over Union (IoU) between the predicted bounding box and the ground-truth bounding box is calculated according to Equation 23:

(23) $$\text{IoU}=\frac{\text{Area}({\text{B}}_{\text{p}}\cap {\text{B}}_{\text{gt}})}{\text{Area}({\text{B}}_{\text{p}}\cup {\text{B}}_{\text{gt}})}$$

Here, B_p_ denotes the predicted bounding box, and B_gt_ denotes the ground-truth bounding box. When IoU ≥ 0.5, the prediction is regarded as a correct detection.

On this basis, the average precision (AP) is calculated for each class, and the mean across all detection classes is taken to obtain mAP_0.5_.This metric comprehensively reflects the overall performance of the model in terms of both flowering panicle localization accuracy and confidence-score ranking.

#### 3.5.3 Model complexity metrics

To evaluate the feasibility of deploying the model on field-based intelligent devices and edge-computing platforms, this study further introduced the following metrics related to model complexity:

Number of parameters (Parameters): Reflects the scale of the model and is closely related to storage overhead and training difficulty.
Computational Cost (GFLOPs): Represents the number of floating-point operations required for a single forward inference and is used to measure inference efficiency.

These metrics can be used to comprehensively evaluate the model’s ability to balance detection accuracy and computational resource consumption, thereby providing an important reference for lightweight deployment in agricultural scenarios.

## 4 Results

### 4.1 Performance evaluation and analysis of YOLO11n-ACDW

To verify the performance advantages of the YOLO11n-ACDW model in rice spikelet flowering-status detection, this study conducted comparative experiments on a self-constructed rice spikelet dataset under the same experimental environment and training strategy. The comparison covered mainstream object detection models, including Faster R-CNN, RT-DETR, and different models in the YOLO series. The experimental results are presented in **Table 2**.

**Table 2:** Detection performance comparison of different models.

Model	P/%	R/%	mAP0.5/%	mAP0.5-0.95/%	Parameter/M	GFLOPs/G
Faster R-CNN	75.8	70.9	76.6	40.9	41.3	136.7
RT-DETR	79.6	78.6	81.5	42.8	20.1	58.6
Yolov3-tiny	70.3	69.2	74.4	39.6	8.67	12.9
Yolov4-tiny	72.1	66.5	71.1	42.1	5.8	7.5
Yolov5n	75.9	68.0	73.4	40.0	2.5	7.1
Yolov6n	75.3	70.7	68	38.3	8.7	11.8
Yolov7-tiny	77.6	78.3	72.1	42.5	6.03	12.3
Yolov8n	80.2	78.9	79.8	43.8	3.01	6.3
Yolov9-tiny	80.1	77.7	75.9	38.6	2.62	10.7
Yolov10n	79.8	80.5	76.1	40.2	2.27	6.5
Yolov11n	80.8	81.3	82.7	41.3	2.58	6.3
Yolo11n-ACDW	88.8	84.7	84.0	48.6	2.14	4.4
Yolov12n	76.0	74.0	80.8	40.7	2.56	5.8

In terms of overall detection performance, YOLO11n-ACDW outperformed the other compared models in the same series. Its precision, recall, and mean average precision reached 88.8%, 84.7% and 84.0%, respectively. Compared with the baseline model YOLO11n, these metrics increased by 8.0%, 3.4% and 1.3%. Compared with the second-best model YOLOv8n, the corresponding improvements were 8.6%, 5.8% and 4.2%. Relative to the traditional two-stage detector Faster R-CNN, the improvements reached 13.0%, 13.8%, and 7.4%.In addition, compared with RT-DETR, YOLO11n-ACDW also showed better performance in terms of precision. These results indicate that the proposed model has stronger feature representation and target discrimination capabilities under complex field-background conditions. From the perspective of computational efficiency, YOLO11n-ACDW had only 2.14 M parameters and a computational cost of 4.4 GFLOPs, which was at a relatively low level among all compared models and was clearly superior to RT-DETR (58.6 GFLOPs) and Faster R-CNN (136.7 GFLOPs). This indicates that the model possesses good lightweight characteristics while maintaining detection accuracy, making it suitable for deployment on agricultural robots and edge-computing devices.

**Figure 8:**
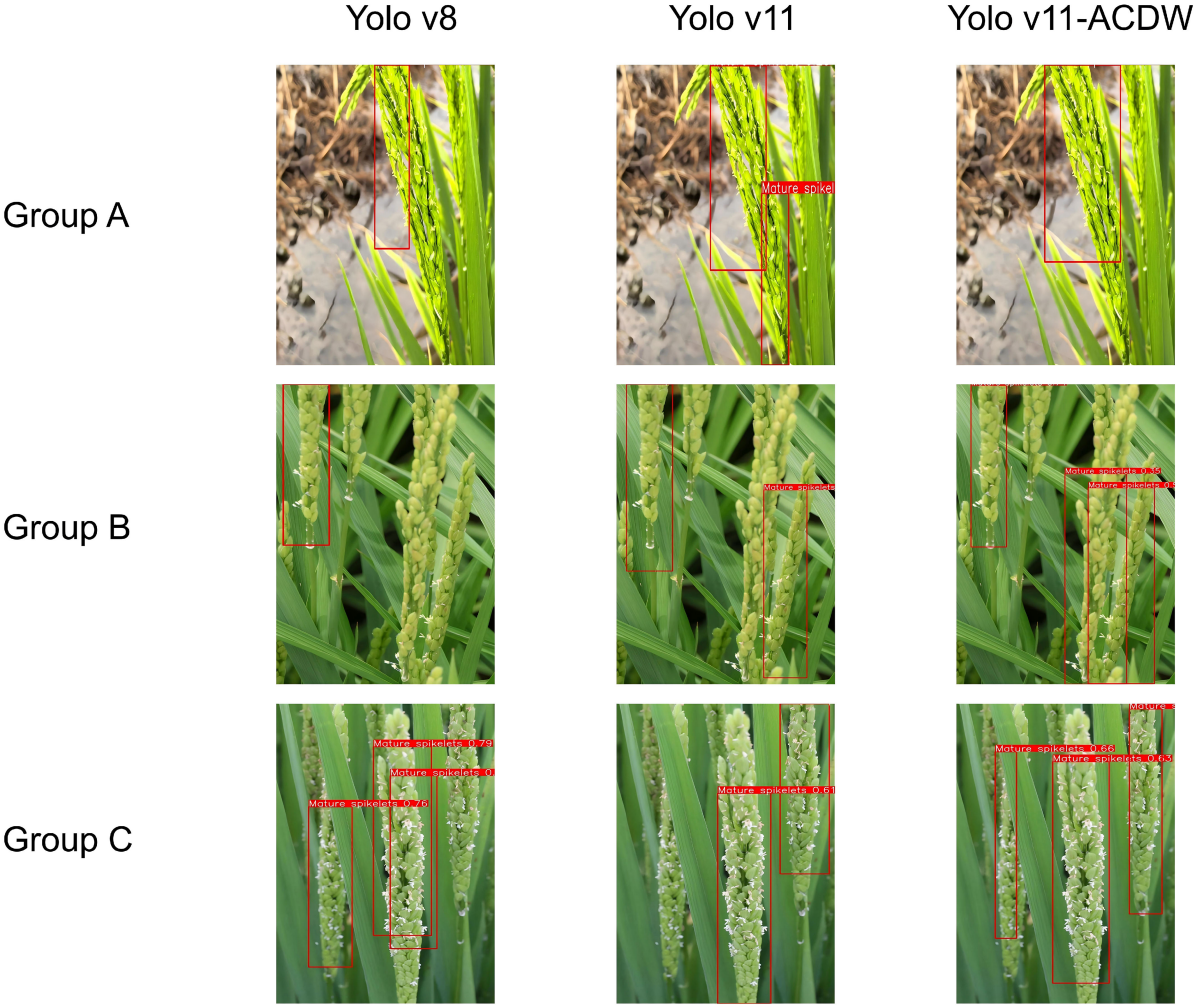
Comparison of detection results from different models.

As further illustrated by the visualization results in **Figure 8**, under complex scenarios, traditional models exhibited both missed detections and false detections in dense panicle regions and under background interference. In contrast, YOLO11n-ACDW was able to identify spikelet regions more completely and showed superior boundary localization capability and robustness, particularly in areas with densely distributed small targets, thereby demonstrating its adaptability to complex field environments.

In summary, YOLO11n-ACDW achieves a favorable balance between detection accuracy and computational efficiency. Compared with existing mainstream models, it shows stronger overall performance and can meet the requirements for high-precision and real-time detection of rice spikelet flowering status.

### 4.2 Ablation study

To verify the performance improvements brought by the enhanced modules in the YOLO11n model, this study conducted a series of ablation experiments to evaluate the effectiveness of each improvement strategy through comparative analysis. The results of the ablation experiments are presented in **Table 3**.

**Table 3:** Ablation study results of different configurations of YOLOv11n-ACDW for rice spikelet flowering-state detection.

Adown module	CAFM Attention Mechanism	Detect_Efficient Detection Head	WiseIoUv3 loss function	P/%	R/%	mAP0.5/%	mAP0.5-0.95/%	GFLOPs/G	Parameter/M
×	×	×	×	80.8 ± 0.21	81.3 ± 0.25	82.7 ± 0.14	41.3 ± 0.28	6.3	2.58
√	×	×	×	84.2 ± 0.22	84.1 ± 0.35	82.9 ± 0.13	46.1 ± 0.26	5.3	2.10
√	√	×	×	83.8 ± 0.14	83.6 ± 0.19	83.2 ± 0.16	44.8 ± 0.30	5.7	2.40
√	√	√	×	85.6 ± 0.24	85.0 ± 0.16	83.3 ± 0.12	47.5 ± 0.32	4.4	2.14
√	√	√	√	88.8 ± 0.18	84.7 ± 0.20	84.0 ± 0.12	48.6 ± 0.25	4.4	2.14

(values reported as mean ± std).

As shown in **Table 3**, compared with the baseline YOLO11n model without any modifications, introducing only the ADown module improved precision, recall, and mean average precision by 3.4%, 2.8%, and 0.2%, while reducing the computational cost to 5.3 GFLOPs and the number of parameters to 2.10 M. These results indicate that the ADown module effectively preserves the feature information of small rice spikelet targets while achieving model lightweighting, thereby laying a foundation for improved detection performance.

Building on this basis, after further incorporating the Convolution–Attention Fusion Module (CAFM), the model achieved 83.8% precision, 83.6% recall, and 83.2% mean average precision. This indicates that CAFM enhances the discrimination between flowering spikelet and complex background regions, such as leaves and rachises, by strengthening the feature response of target regions. After the further introduction of the Detect_Efficient detection head, the computational cost was reduced to 4.4 GFLOPs while the number of parameters remained at a lightweight level, demonstrating the suitability of this detection head for rice spikelet detection. When all improved modules were integrated and the Wise-IoU v3 loss function was introduced, the model precision and mean average precision reached 88.8% and 84.0%, respectively, representing improvements of 8.0% and 1.3% over the corresponding metrics of the baseline model. Meanwhile, the computational cost and number of parameters still remained at a low computational and parameter level. This suggests that the Wise-IoU v3 loss function forms effective synergy with the preceding modules and enhances the stability and accuracy of bounding-box regression.

**Figure 9:**
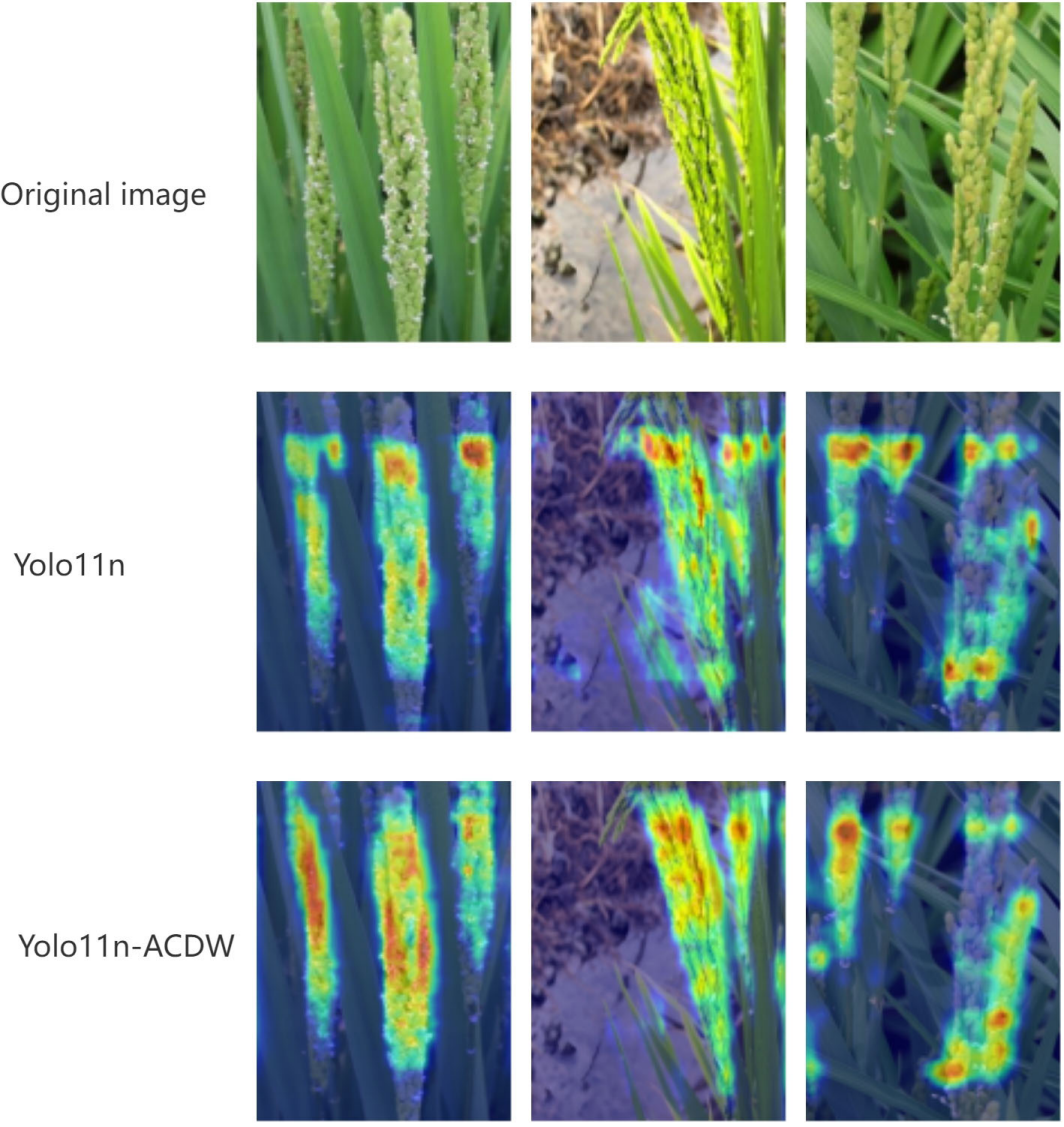
Visual comparison of ablation models on the same rice panicle areas.

To further illustrate the contribution of each module, paired detection screenshots of the same rice panicle area were generated under identical prediction settings. As shown in **Figure 9**, the baseline YOLO11n model detected only a limited number of flowering spikelet and missed several targets in dense spikelet regions. After introducing ADown, more small spikelet targets were detected, indicating improved small-target feature preservation. The addition of CAFM enhanced detection stability under complex leaf and rachis backgrounds. Detect_Efficient further improved detection completeness in densely distributed spikelet regions. After introducing Wise-IoU v3, the bounding boxes became more stable and better aligned with flowering spikelet regions. These visual results are consistent with the quantitative ablation results in **Table 3**.

Overall, the ablation experiments demonstrate that the proposed improvement strategy provides hierarchical optimization at different levels, including feature preservation, foreground-background discrimination, multi-scale feature allocation, and bounding-box regression. Through the synergistic effects of these modules, YOLO11n-ACDW reduces missed detections and improves localization performance while maintaining a lightweight model structure.

### 4.3 Comparison of loss functions

To validate the effectiveness of the WIoUv3 loss function, we compared it with the CIoU, DIoU, EIoU, SIoU, NWD, and MPDIoU loss functions, as well as the Focal-CIoU and Inner-CIoU loss functions, which are optimized for specific scenarios. The results are shown in **Table 4**.

**Table 4:** Detection performance comparison of different loss functions.

IoU loss	P/%	R/%	mAP0.5/%	mAP0.5-0.95/%	Parameter/M	GFLOPs/G
CIoU	85.6	85	83.3	47.5	2.14	4.4
DIoU	85.2	79.4	82.2	44.3	2.14	4.4
EIoU	82.7	80.3	81.5	48	2.14	4.4
SIoU	87.6	88	83.1	47.6	2.14	4.4
NWD	83.4	81.3	80.4	49.6	2.14	4.4
MPDIoU	76.6	79.3	78	47.5	2.14	4.4
Focal-CIoU	81.1	81.2	80.2	47	2.14	4.4
Inner-CIoU	87.4	79.3	80.6	48.7	2.14	4.4
WIoU v3	88.8	84.7	84	48.6	2.14	4.4

As shown in **Table 4**, when the YOLO11n-ACDW model uses the WIoUv3 loss function, mean average precision improves by 0.7% compared with the baseline CIoU loss function. WIoUv3 also shows significant performance advantages over other loss functions. When compared with scenario-specific optimizations such as Focal-CIoU, Inner-CIoU, and NWD, WIoUv3’s superiority in mAP further validates the effectiveness of its design. Focal-CIoU improves detection performance by introducing a focal loss mechanism, but its performance is still inferior to WIoUv3. Inner-CIoU penalizes areas inside the bounding box and is less sensitive to boundary deviations for elongated objects. In contrast, WIoUv3 dynamically adjusts loss weights to balance gradient stability for easy samples and optimization for difficult samples. This provides stable gradients even in cases with uneven target size distributions and thus performs better on small and unusually shaped objects. NWD shows advantages in handling very small objects and performs well on the mAP@0.5:0.95 metric. However, it still falls short of WIoUv3 in overall detection accuracy and gradient stability.

In summary, the WIoUv3 loss function used in this study has demonstrated significant performance advantages in comparative experiments and effectively improved object detection accuracy.

### 4.4 Model interpretability analysis

To visually verify the feature-focusing capability of the YOLO11n-ACDW improvement strategy for small rice spikelet targets, this study employed the Grad-CAM method to capture the output features of the final convolutional layer of the model and generate class activation heatmaps (**Figure 9**) (Selvaraju et al., 2020). In the heatmaps, color intensity indicates the degree of feature attention paid by the model to the corresponding regions. Regions closer to red indicate that the model shows stronger capability in identifying and focusing on target features in those areas.

**Figure 10:**
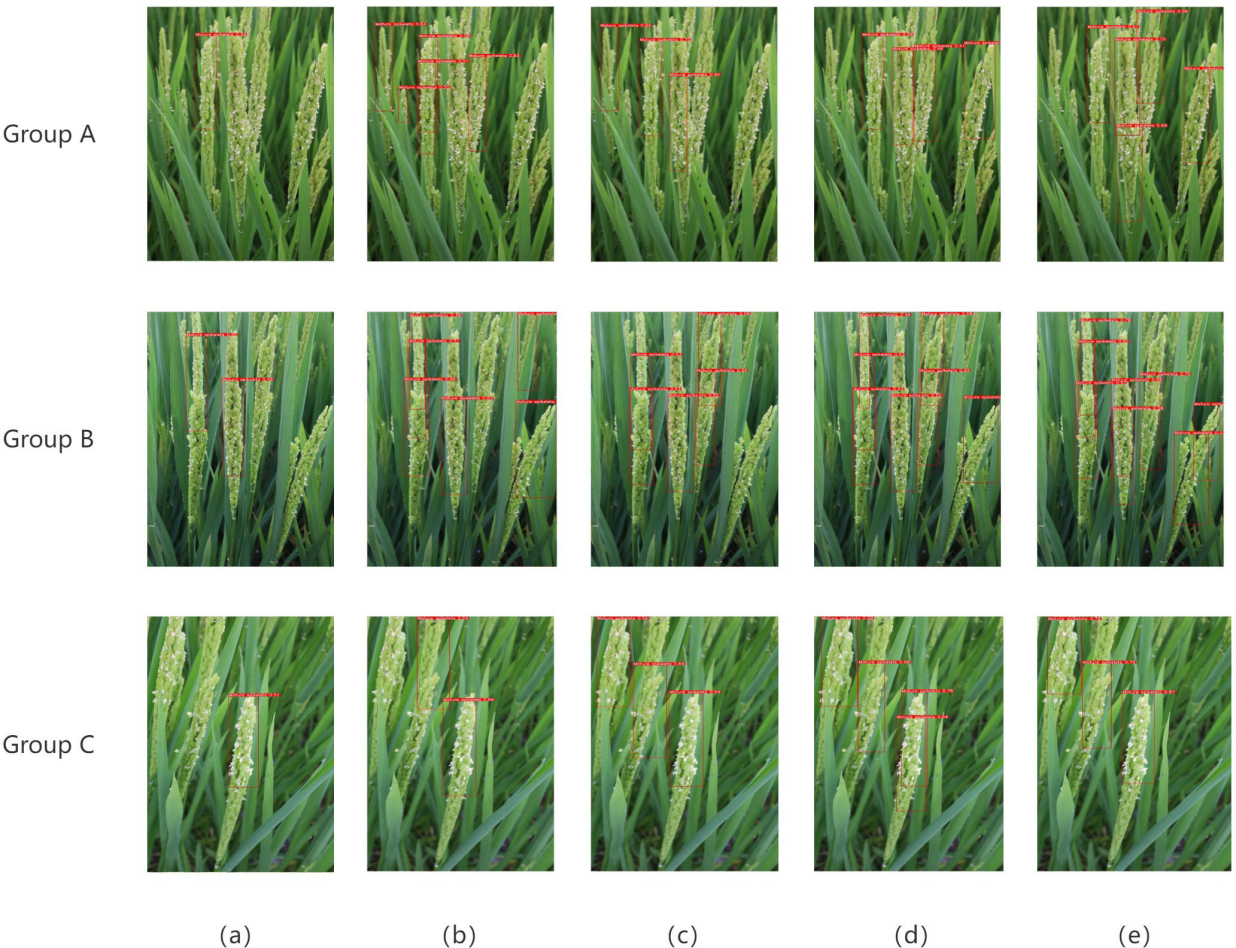
Comparison of heatmap results.

**Figure 10** presents a comparison of the original image and the heatmaps generated by YOLO11n and YOLO11n-ACDW under a densely planted field rice scenario. It can be observed that the heatmap produced by the baseline model YOLO11n shows a relatively scattered attention distribution. Although some panicle regions are covered, invalid activations are still present in background regions such as leaves and stems, indicating that the model fails to precisely focus on the small-target features of flowering rice spikelet. In contrast, the heatmap generated by YOLO11n-ACDW exhibits more concentrated and accurate activation regions. The red-highlighted areas closely correspond to the locations of flowering rice spikelet. Even under conditions of dense panicles and strong background interference, the improved model can still effectively distinguish targets from the background, substantially reducing feature activation in irrelevant regions.

These visualization results indicate that the improvement strategy can specifically enhance the model’s attention to the features of small rice spikelet targets. By suppressing interference from redundant background information, the model is better able to focus on the effective features of flowering rice spikelet, thereby improving the accuracy of small-target detection in complex densely planted field scenarios.

### 4.5 Analysis of detection results under different fusion ratios

Although single-stage YOLO models have the advantages of high speed and convenient deployment, under complex field-background conditions, the features of rice spikelet, which are extremely small, densely distributed, and have indistinct boundaries, are easily overwhelmed by background information, leading to missed detections, false detections, and inaccurate localization. Therefore, this study proposed a data processing workflow consisting of image segmentation, panicle region-of-interest (ROI) extraction, and spikelet annotation. To verify the effect of the fusion ratio between original sub-images and panicle ROI images on detection performance, multiple data-fusion schemes with different ratios were designed while keeping the total number of training samples unchanged. These schemes included combinations of 1:0, 1:1, 1:2, 1:3, 2:1, and 2:3 (original images: panicle ROI images). All fusion schemes were based on the same image-segmentation method, panicle-mask construction method, and flowering-panicle detection labels. The YOLO11n-ACDW model was used for testing, and comparative experiments were conducted under the same training parameters. During the testing stage, original images were used for evaluation to further verify the generalization ability and practical value of this strategy in real complex field environments.

The experimental results are summarized in **Table 5**. The results indicate that when the original images and panicle ROI images were fused at a ratio of 1:1, the model achieved the best performance in terms of precision, recall, and mean average precision. This ratio effectively suppresses background interference while preserving necessary contextual information, thereby enabling the model to achieve more stable localization and discrimination of flowering panicles in complex field environments. In contrast, when the proportion of panicle ROI images is too high, the model’s ability to judge panicle scale and spatial relationships decreases; when the proportion of original images is too high, background interference remains pronounced.

**Table 5:** Detection results under different fusion ratios.

Data ratio	P/%	R/%	mAP0.5/%	mAP0.5-0.95/%
1:0	76.2	78.7	77.6	43.7
1:1	88.8	84.7	84.0	48.6
1:2	85.1	80.7	82.3	46.1
2:1	84.2	81.3	79.7	45.3
3:1	78.3	74.0	78.3	44.2
3:2	79.7	76.2	81.0	45.6

Based on the above results, the reasonable fusion of original images and panicle ROI images can achieve good detection performance and provide the YOLO11n-ACDW model with a discriminative input dataset.

## 5 Conclusion

This study proposed a dataset construction strategy and a lightweight detection model, YOLO11n-ACDW, for small-target rice spikelet detection in hybrid rice breeding. By integrating raw field images with panicle ROI information, structured foreground constraints were introduced to enhance the representation of spikelet regions under complex field conditions. Experimental results showed that the proposed model achieved precision, recall, and mAP@0.5 of 88.8%, 84.7%, and 84.0%, respectively, while maintaining a low parameter count and computational cost. These results indicate that YOLO11n-ACDW can effectively improve rice spikelet detection performance and provide a feasible technical solution for intelligent monitoring during the rice flowering period.

**Figure 11:**
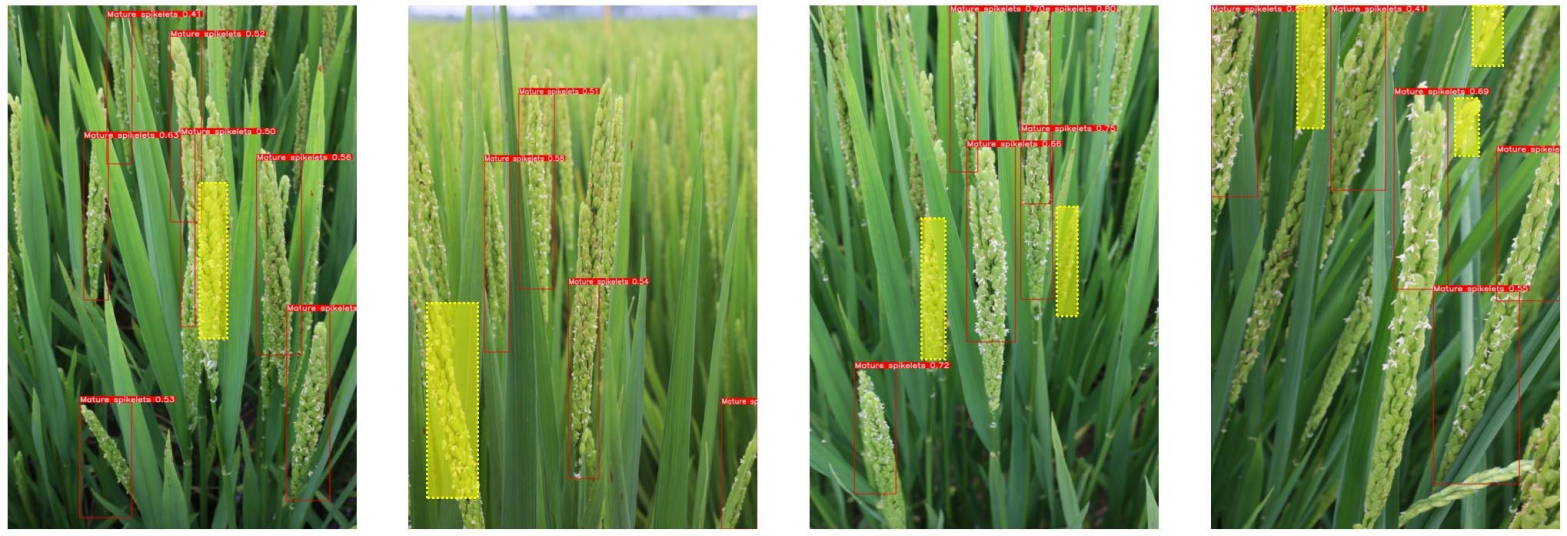
Typical missed detection cases of rice spikelet.

Nevertheless, several limitations remain. Missed detections still occur under extreme illumination, severe occlusion, and dense spikelet distribution, as shown in **Figure 11**. In addition, the training data were collected mainly from specific experimental sites and a limited number of rice varieties, and the practical deployment of the model on field equipment has not yet been systematically evaluated. Future work will focus on expanding the dataset across more varieties and ecological regions, improving model robustness under complex field conditions, and exploring a two-stage framework that combines semantic segmentation with object detection. Lightweight network design, model compression, and the incorporation of multispectral or hyperspectral information will also be considered to support low-power real-time detection on embedded platforms.

## Data Availability

The original contributions presented in the study are included in the article/supplementary material. Further inquiries can be directed to the corresponding author.
